# Superior Vena Cava Syndrome and Pulmonary Embolism Revealing a Lung Sarcomatoid Carcinoma: An Uncommon Presentation of a Rare Tumor

**DOI:** 10.7759/cureus.117503

**Published:** 2026-10-05

**Authors:** Amina Chadli, Soufiane Berhili, Ahmed BenSghier, Mohamed Moukhlissi, Loubna Mezouar

**Affiliations:** 1 Radiation Oncology, Faculty of Medicine and Pharmacy, Mohammed First University, Oujda, MAR; 2 Radiation Oncology, Centre Hospitalier Universitaire (CHU) Mohammed VI, Oujda, MAR; 3 Radiotherapy, Mohammed VI University Hospital, Oujda, MAR

**Keywords:** metastatic desease, non-small cell lung cancer, pulmonary sarcomatoid carcinoma, superior vena cava syndrome, tobacco smoking

## Abstract

Pulmonary sarcomatoid carcinoma (PSC) is a rare and highly aggressive subtype of non-small cell lung cancer, associated with early metastatic spread and poor prognosis. Tumor-related thromboembolic complications and superior vena cava (SVC) syndrome are uncommon but severe manifestations.

We report the case of a 62-year-old man with a history of smoking and alcohol use, presenting with a large right upper lobe tumor with widespread metastases, associated with radiologically suspected tumor-related pulmonary embolism and SVC invasion with multiple venous thromboses. Bronchoscopic biopsy with immunohistochemical analysis confirmed PSC. The patient received anticoagulation and urgent palliative radiotherapy (20 Gy in five fractions) for brain metastases and SVC syndrome. This case highlights the aggressive nature of PSC, its association with rare vascular complications, and the challenges of emergency palliative management in patients with advanced disseminated disease.

## Introduction

Pulmonary sarcomatoid carcinoma (PSC) is a rare and highly aggressive subtype of non-small cell lung cancer (NSCLC), accounting for approximately 0.1%-0.4% of all lung malignancies [1]. Histologically, PSC is characterized by the coexistence of epithelial and sarcomatoid components and is associated with rapid tumor progression, marked invasiveness, and poor clinical outcomes [2].

PSC predominantly affects older male smokers and typically presents with nonspecific respiratory symptoms, including cough, hemoptysis, and bloody sputum. The primary tumor most commonly arises in the upper lobes, particularly the right upper lobe. At diagnosis, many patients present with locally advanced or metastatic disease. Common metastatic sites include the lungs, brain, liver, adrenal glands, and bone, whereas less frequent metastatic locations, such as the peritoneum, pancreas, kidney, skin, and right ventricle, have also been reported in advanced-stage PSC [3,4].

The prognosis of PSC remains significantly poorer than that of other NSCLC subtypes, with a median overall survival ranging from eight to 19 months and a reported five-year survival rate of only 17%-29% [4]. Advanced tumor stage, lymph node involvement, and distant metastasis have been identified as the principal adverse prognostic factors [5].

We report a histologically confirmed case of metastatic PSC presenting with malignant superior vena cava (SVC) syndrome and radiologically suspected tumor-related pulmonary embolism. This case highlights the aggressive clinical behavior of PSC and the challenges of emergency palliative management in patients with advanced metastatic disease.

## Case presentation

A 62-year-old man with a 24-pack-year smoking history who had quit smoking 20 years earlier and a history of chronic alcohol consumption presented with a progressively worsening productive cough and recurrent hemoptysis associated with marked general health deterioration. Over the following weeks, he developed resting dyspnea, dizziness, facial swelling, and progressive edema of the left upper limb, raising clinical suspicion of malignant SVC syndrome.

On admission, the patient appeared chronically ill but remained hemodynamically stable. Physical examination revealed facial swelling and left upper-limb edema, without jugular venous distension, facial cyanosis, or visible collateral venous circulation. The patient reported no headaches or seizures. His Eastern Cooperative Oncology Group (ECOG) performance status was 3 [6]. Vital signs and relevant laboratory findings at admission are summarized in Table 1. The observed tachycardia and tachypnea were consistent with the severity of the patient's acute cardiopulmonary presentation. The laboratory assessment was notable for microcytic hypochromic anemia and a markedly elevated C-reactive protein level, reflecting a pronounced systemic inflammatory response, although its underlying cause could not be definitively determined. The etiology of the anemia was not further investigated during the patient's rapidly evolving clinical course. Urine output was preserved.

**Table 1:** Vital signs and laboratory findings at admission

Parameter	Patient value	Unit	Reference range
Blood pressure	150/80	mmHg	90-120/60-80
Heart rate	115	beats/min	60-100
Respiratory rate	25	breaths/min	12-20
Peripheral oxygen saturation (SpO₂), room air	93	%	95-100
Body temperature	37	°C	36.5-37.5
Hemoglobin	10.4	g/dL	13-17
Mean corpuscular volume (MCV)	76.3	fL	82-98
C-reactive protein	183	mg/L	<6
White blood cell count	7930	/mm³	4000-10000
Platelet count	305000	/mm³	150000-400000
Prothrombin time	83	%	70-100

Contrast-enhanced computed tomography (CT) of the chest revealed a large heterogeneous right upper lobe mass measuring 105 × 103 × 85 mm, containing central necrotic areas and extending to the right hilum and carina (Figure 1).

**Figure 1:**
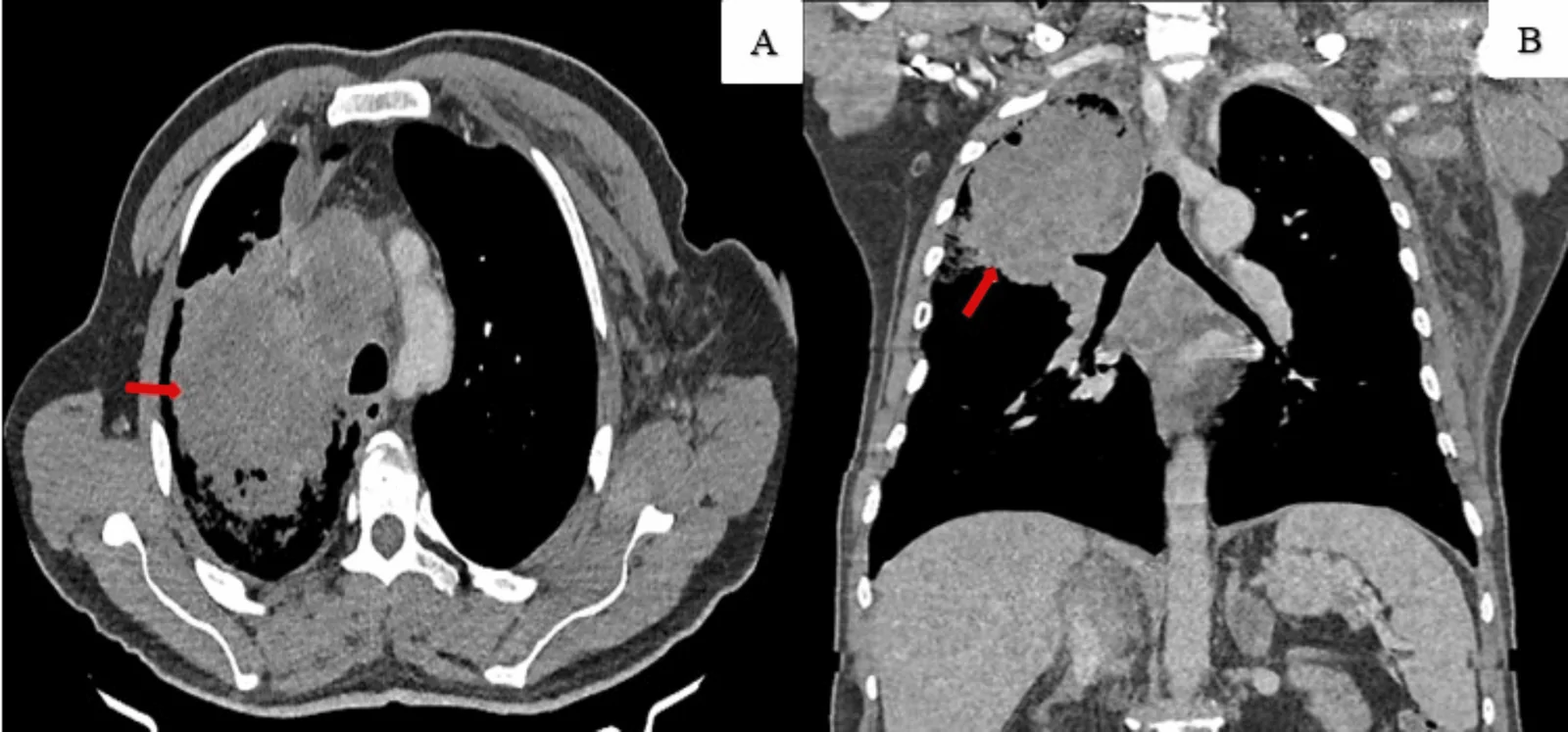
Contrast-enhanced chest CT images demonstrating the primary pulmonary tumor (A) Axial image showing a bulky heterogeneous right upper lobe mass with areas of necrosis, measuring 105 × 103 × 85 mm (red arrow). (B) Coronal reconstruction demonstrating the extent of the tumor and its extension toward the right hilum and carina (red arrow)

The lesion encased the right upper lobar bronchus and invaded the right upper lobar pulmonary artery. Multiple enlarged mediastinal, bilateral hilar, and left supraclavicular lymph nodes were identified. Mediastinal nodal disease invaded the SVC and the brachiocephalic (innominate) vein, with associated thrombosis of the left subclavian and left internal jugular veins, consistent with malignant SVC syndrome (Figure 2).

**Figure 2:**
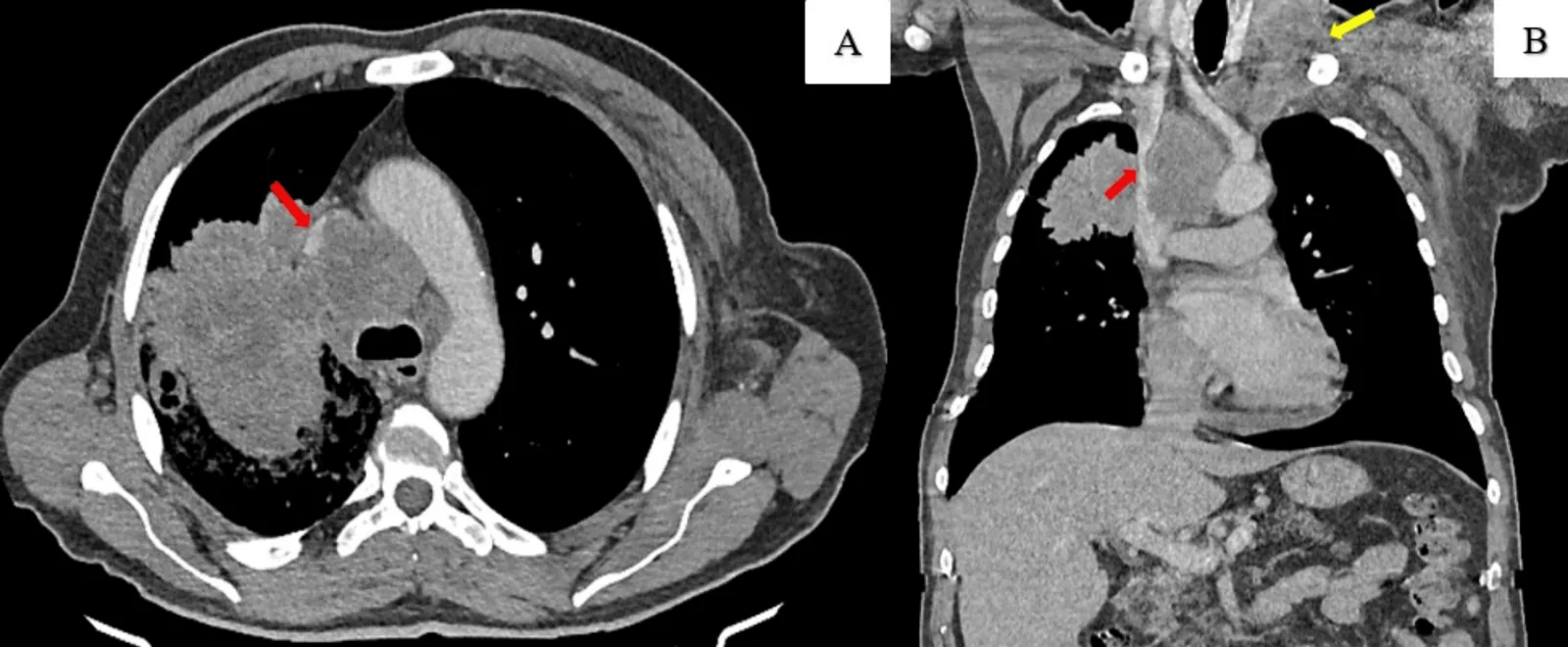
Contrast-enhanced chest CT images demonstrating malignant superior vena cava syndrome (A) Axial and (B) coronal views showing extensive mediastinal tumor involvement with invasion of the superior vena cava (red arrows). The yellow arrow indicates left supraclavicular lymphadenopathy

Contrast-enhanced chest CT additionally demonstrated a proximal right pulmonary arterial filling defect radiologically suspicious for tumor-related pulmonary embolism (Figure 3).

**Figure 3:**
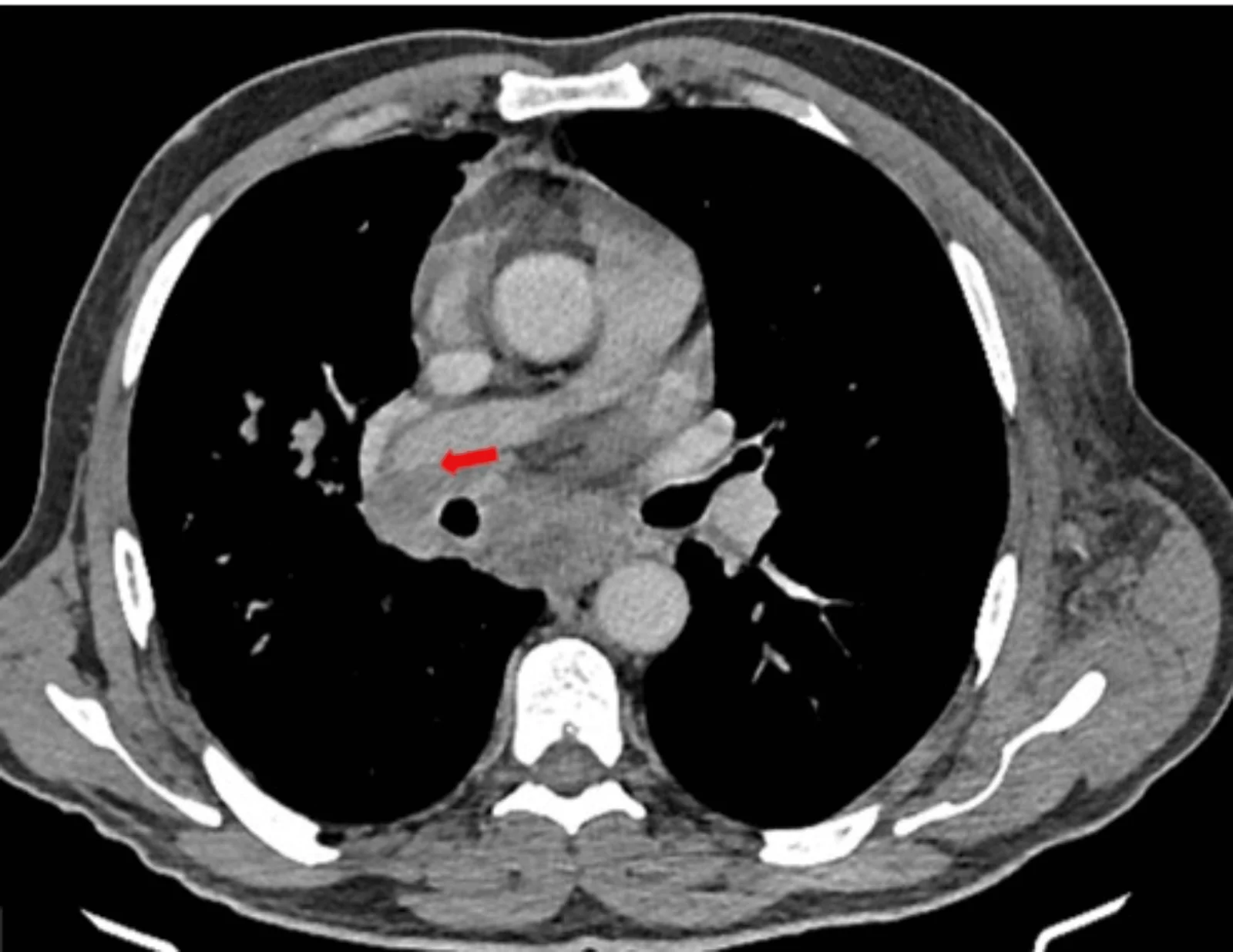
Contrast-enhanced axial chest CT demonstrating a proximal filling defect within the right pulmonary artery (red arrow), interpreted radiologically as suspicious for tumor-related pulmonary embolism

A moderate pericardial effusion measuring 17 mm adjacent to the right ventricle and a minimal right pleural effusion were also observed. Further staging investigations demonstrated widespread metastatic disease involving both lungs, the liver (including a segment V lesion measuring 39 × 36 mm), and both adrenal glands. Multiple peritoneal nodules consistent with peritoneal carcinomatosis were also identified (Figure 4), along with a suspicious left iliac bone lesion. Brain CT revealed two posterior parietal cortico-subcortical lesions, measuring 14 × 14 mm on the right and 4 × 3 mm on the left, consistent with brain metastases. The larger right-sided lesion was associated with surrounding vasogenic edema (Figure 5). No significant midline shift or hydrocephalus was observed.

**Figure 4:**
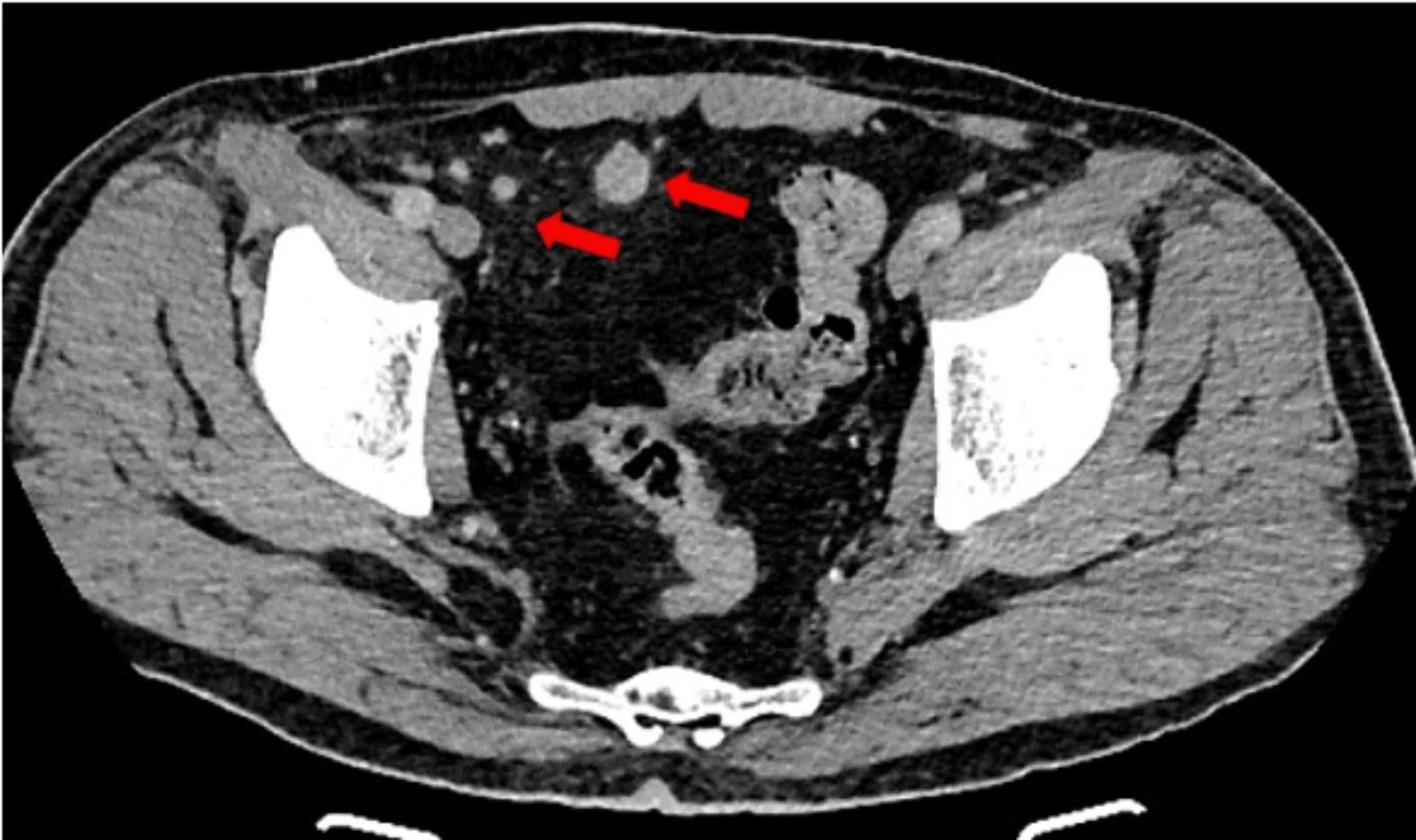
Contrast-enhanced axial abdominopelvic CT demonstrating peritoneal nodules (red arrows) consistent with peritoneal carcinomatosis

**Figure 5:**
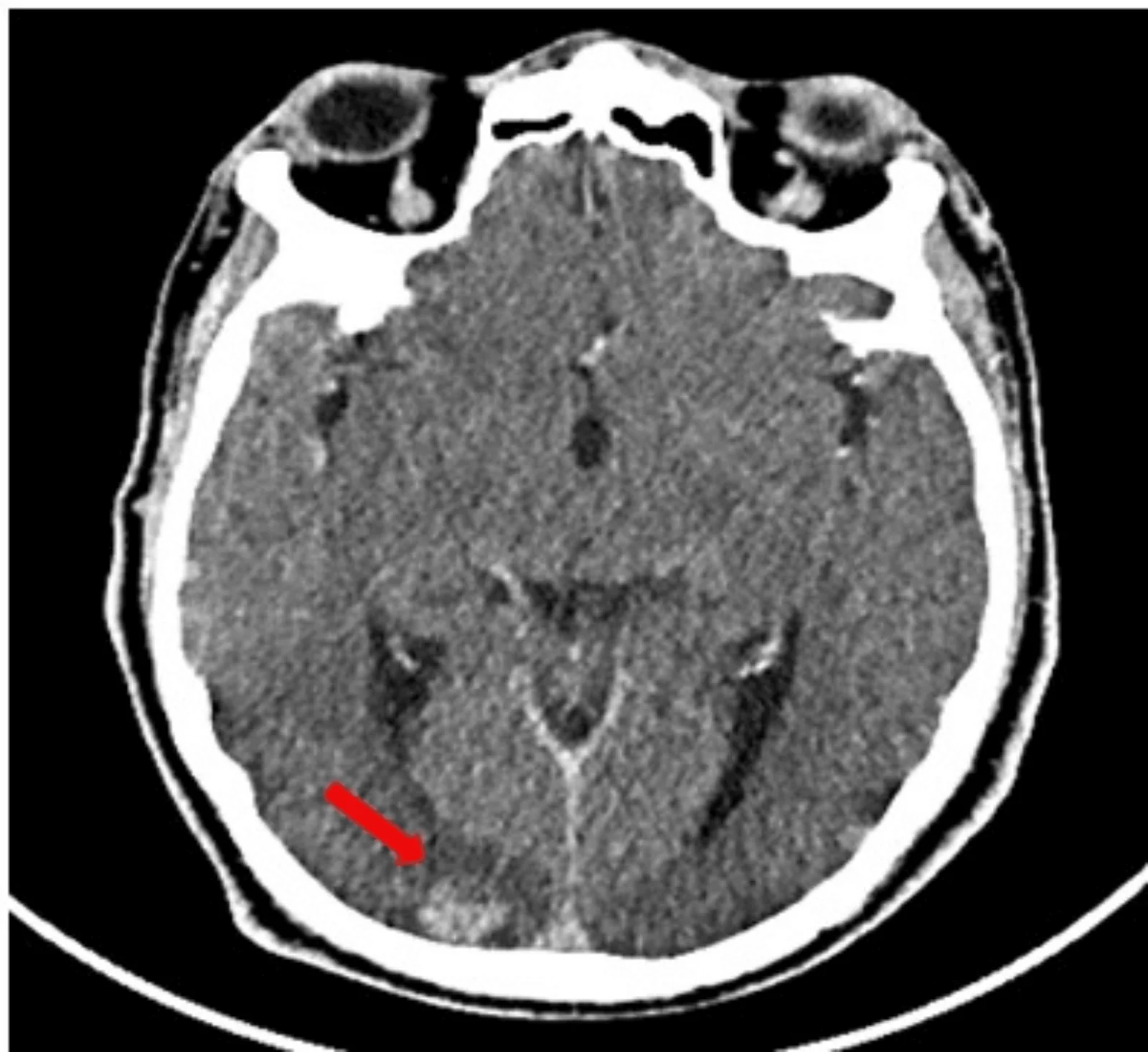
Contrast-enhanced axial brain CT demonstrating the larger of two posterior parietal cortico-subcortical metastatic lesions, located on the right and measuring 14 × 14 mm, with surrounding vasogenic edema (red arrow)

Flexible bronchoscopy revealed an endobronchial lesion involving the right upper lobe bronchus. Histopathological examination of seven bronchoscopic biopsy fragments demonstrated diffuse infiltration of the bronchial mucosa by a poorly differentiated malignant neoplasm composed of large pleomorphic cells with marked nuclear atypia, coarse chromatin, prominent nucleoli, abundant basophilic cytoplasm, and frequent mitotic figures (Figures 6A-6B).

**Figure 6:**
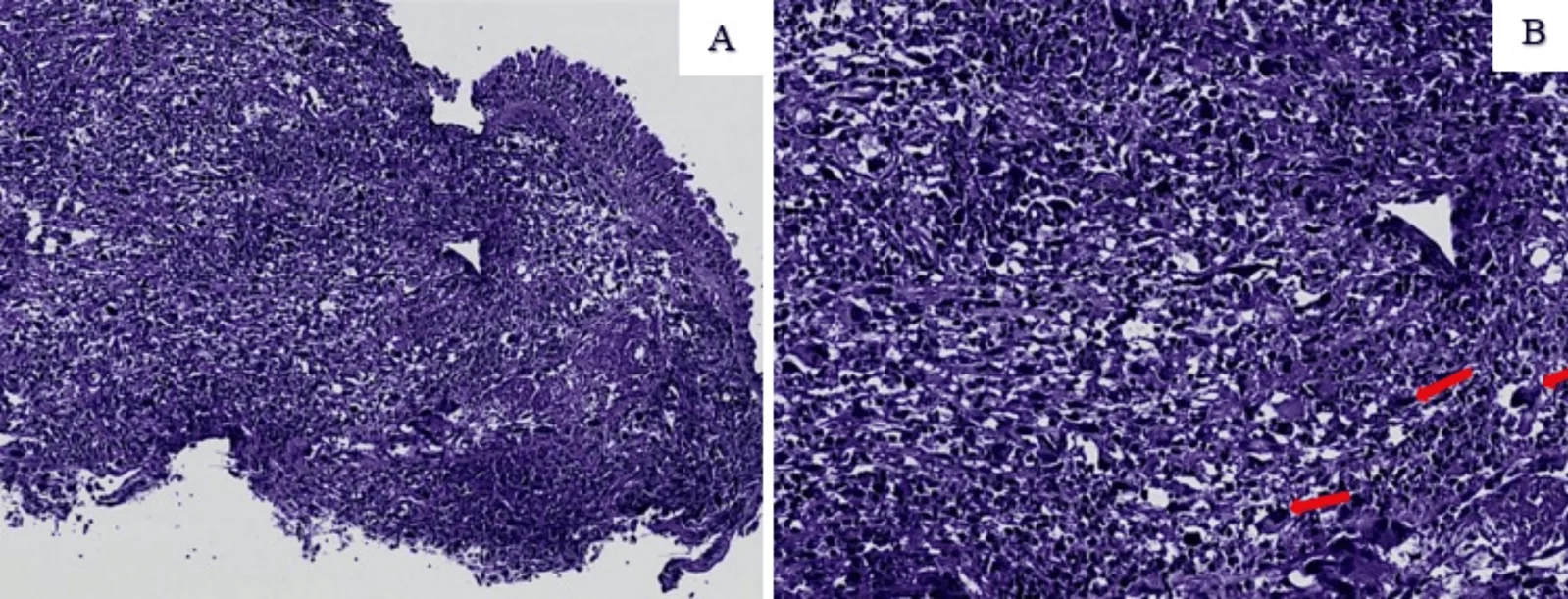
Histopathological examination of the bronchoscopic biopsy (A) Hematoxylin and eosin (H&E) staining (X10) showing diffuse infiltration of the bronchial mucosa by a poorly differentiated malignant neoplasm. (B) Higher-power view (X20) demonstrating large pleomorphic tumor cells with marked nuclear atypia, conspicuous nucleoli, abundant eosinophilic to basophilic cytoplasm (red arrows)

No evidence of glandular differentiation, squamous differentiation, lymphovascular invasion, or perineural invasion was identified. Immunohistochemical analysis showed diffuse positivity for AE1/AE3 (pan-cytokeratin) and focal weak positivity for CK7, while CK20, TTF-1, P40, PAX8, Hepatocyte Ab-1, PSA, synaptophysin, and chromogranin A were negative. Alcian blue staining was also negative (Figure 7).

**Figure 7:**
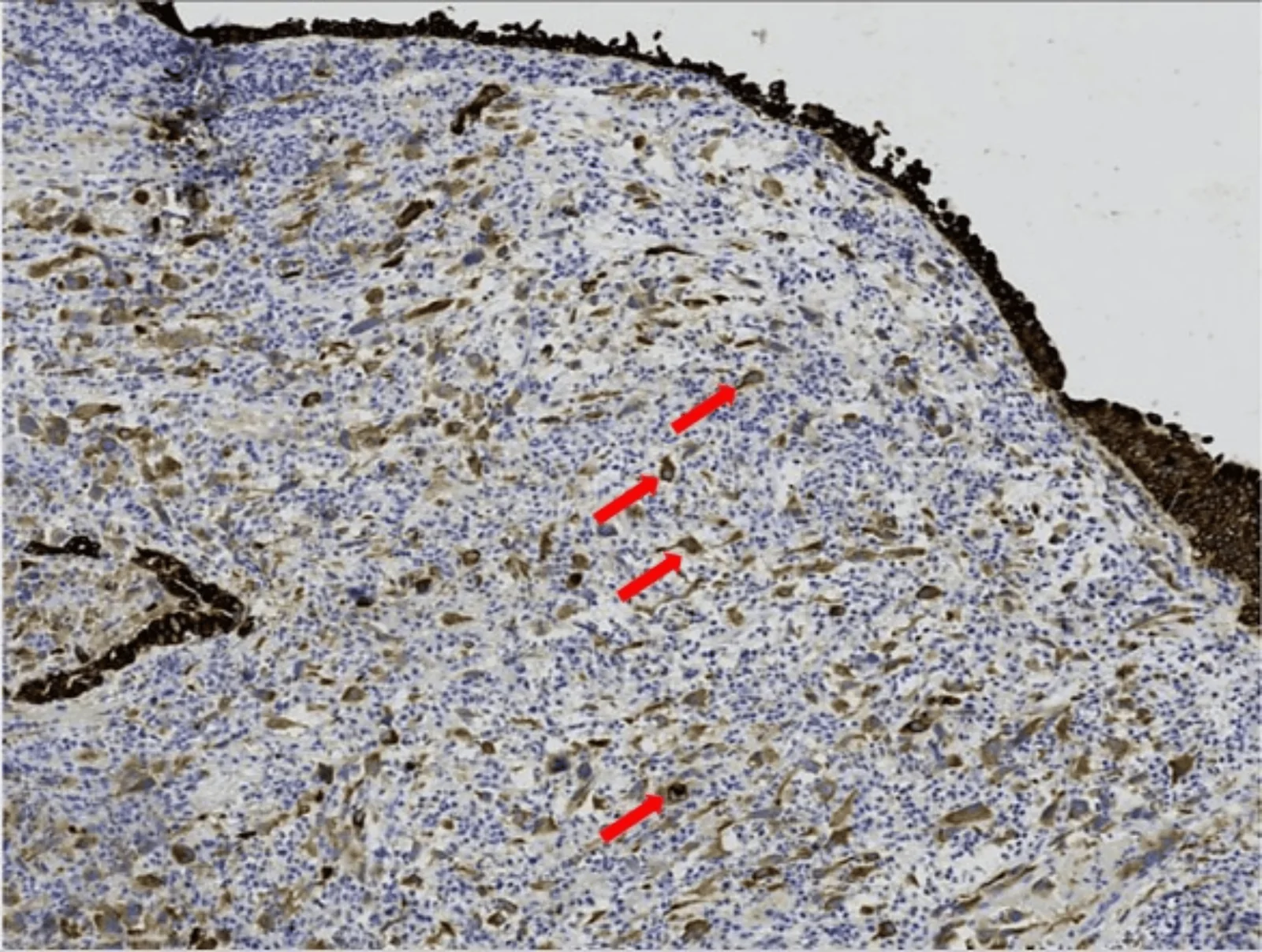
Immunohistochemical staining demonstrating diffuse cytoplasmic positivity for pan-cytokeratin (AE1/AE3) (red arrows), confirming epithelial differentiation In conjunction with the histomorphological findings and the complete immunophenotypic profile, these results supported the diagnosis of pulmonary sarcomatoid carcinoma

The diffuse expression of pan-cytokeratin confirmed epithelial differentiation, whereas the absence of lineage-specific markers excluded alternative differential diagnoses, including conventional pulmonary adenocarcinoma, squamous cell carcinoma, neuroendocrine carcinoma, and metastatic tumors from extrapulmonary primary sites. Considering the histomorphological findings, the complete immunophenotypic profile, and the clinicoradiological presentation, the diagnosis of PSC was established.

Taken together, the clinical, radiological, histopathological, and immunohistochemical findings established the diagnosis of metastatic PSC complicated by malignant SVC syndrome and radiologically suspected tumor-related pulmonary embolism. Given the disseminated metastatic disease, poor performance status, and the coexistence of two oncologic emergencies-malignant SVC syndrome and brain metastases-the patient was considered unsuitable for curative treatment. High-dose corticosteroids and therapeutic anticoagulation were initiated, followed by emergency palliative radiotherapy. Whole-brain irradiation and mediastinal radiotherapy targeting the SVC syndrome were simultaneously planned using a conventional two-dimensional technique to a total dose of 20 Gy in five daily fractions (4 Gy per fraction). Despite prompt initiation of supportive measures and emergency palliative radiotherapy, the patient's clinical condition continued to deteriorate rapidly, and he died shortly after receiving the second fraction of palliative radiotherapy.

## Discussion

PSC is a rare and highly aggressive subtype of non-small cell lung cancer (NSCLC), accounting for approximately 0.1%-0.4% of malignant pulmonary tumors [3,7]. According to the World Health Organization classification, this heterogeneous group comprises poorly differentiated carcinomas displaying sarcomatous or sarcoma-like differentiation, including pleomorphic carcinoma, spindle cell carcinoma, giant cell carcinoma, carcinosarcoma, and pulmonary blastoma [7,8]. PSC predominantly affects older adults, particularly men with a history of tobacco exposure, and is characterized by rapid growth, early metastatic dissemination, and poorer outcomes than conventional NSCLC [3,9].

The pathological diagnosis of PSC can be challenging, particularly when it is based on a small bronchoscopic biopsy. The sarcomatoid component may be heterogeneous and inadequately represented in limited tissue samples, while the marked pleomorphism may overlap with primary pulmonary sarcoma, metastatic sarcoma, melanoma, mesothelioma, and other poorly differentiated carcinomas. Immunohistochemistry is therefore used to demonstrate epithelial differentiation and exclude alternative diagnoses. Cytokeratin expression may be diffuse, focal, or occasionally weak in the sarcomatoid component; consequently, a panel of epithelial markers is preferable to reliance on a single stain [8,10]. In the present case, diffuse pan-cytokeratin AE1/AE3 expression confirmed epithelial differentiation, while focal CK7 positivity and the absence of TTF-1, p40, neuroendocrine markers, and markers suggesting selected extrapulmonary primary sites supported the pathologist’s diagnosis when interpreted together with the morphology and pulmonary presentation.

Nevertheless, pan-cytokeratin positivity is not specific for PSC and does not establish this diagnosis independently. It confirms that the malignant cells possess epithelial differentiation, thereby favoring a carcinoma over a purely mesenchymal sarcoma. Likewise, negative TTF-1 and p40 staining does not exclude PSC because sarcomatoid tumors may lose the lineage-specific markers expressed by more differentiated adenocarcinomas or squamous cell carcinomas [8,10]. The diagnosis should therefore be presented as an integrated conclusion based on histomorphology, immunophenotype, and clinicoradiological correlation rather than as the result of AE1/AE3 positivity alone.

PSC is frequently diagnosed at a locally advanced or metastatic stage. In a retrospective series of 55 patients, approximately 60% had locally advanced or metastatic disease at diagnosis, and the reported median overall survival was approximately 12 months [9]. The lungs, brain, liver, adrenal glands, and bones are among the recognized sites of distant dissemination [3]. The present patient had a particularly extensive disease burden involving both lungs, the brain, liver, bilateral adrenal glands, peritoneum, and a suspected iliac bone site. Peritoneal dissemination is less commonly described than cerebral, hepatic, adrenal, pulmonary, or skeletal involvement and, in this context, further reflected the disease's markedly aggressive course.

The remarkable aggressiveness of PSC is thought to be largely driven by epithelial-mesenchymal transition (EMT), a biological process that promotes tumor invasion, vascular infiltration, early metastatic dissemination, and resistance to conventional chemotherapy and radiotherapy. These biological characteristics help explain why most patients present with advanced disease and experience poor outcomes despite multimodal treatment [11].

Although PSC is well-recognized for its aggressive behavior and early metastatic dissemination, the simultaneous occurrence of multiple oncologic emergencies at initial presentation remains uncommon. A recently published case by Patel et al. described PSC presenting with malignant SVC syndrome secondary to mediastinal invasion, further complicated by pericardial metastasis, pleural effusions, hypoxic respiratory failure, and atrial fibrillation. However, unlike our patient, pulmonary tumor embolism and extensive venous thrombosis were not reported. The coexistence of malignant SVCS, extensive venous thrombosis, radiologically suspected pulmonary tumor embolism, synchronous brain metastases, and peritoneal dissemination in our patient therefore represents an even more complex presentation of disseminated PSC [12].

Another important feature of this case was malignant SVC syndrome. Malignant SVCS results from obstruction of the SVC by external compression, direct tumor invasion, intraluminal thrombosis, or a combination of these mechanisms [13,14]. Thoracic malignancies, particularly lung cancer, remain major causes of malignant SVCS [13]. Impaired venous return from the head, neck, and upper extremities commonly causes facial or neck swelling, upper-limb edema, venous distension, dyspnea, cough, or, in severe cases, neurological symptoms [13,14]. In our patient, facial swelling and left upper-limb edema were explained by direct invasion of the SVC and brachiocephalic veins, together with thrombosis of the left internal jugular and subclavian veins.

The CT examination also identified a proximal right pulmonary arterial filling defect that was interpreted by the radiologist as being of tumor origin. Pulmonary tumor embolism is caused by embolization of malignant cells or tumor fragments into the pulmonary arterial circulation and is distinct from conventional fibrin-rich venous thromboembolism. It is difficult to diagnose before death because its clinical and radiological findings may resemble conventional pulmonary thromboembolism, and microscopic tumor emboli may not be visible on CT pulmonary angiography [15,16]. Definitive confirmation generally requires histological demonstration of tumor cells within the pulmonary vasculature or examination of retrieved embolic material [15]. Therefore, in the absence of pathological confirmation, the pulmonary arterial filling defect was considered radiologically suspicious for tumor-related pulmonary embolism.

This distinction also has therapeutic implications. Anticoagulation is standard treatment for conventional cancer-associated venous thromboembolism, but isolated tumor emboli may not respond in the same manner because the obstruction contains malignant tissue rather than only thrombotic material [15,16]. Tumor-directed treatment is therefore central whenever the patient’s condition and tumor sensitivity permit it. However, concomitant fibrin-rich thrombosis may coexist with tumor invasion, particularly in patients with extensive venous thrombosis, and anticoagulation may therefore remain appropriate after an individualized assessment of thrombotic and bleeding risks [17]. In the present case, the associated thrombosis of the left internal jugular and subclavian veins provided an additional conventional thrombotic indication for therapeutic anticoagulation, although recurrent hemoptysis required careful evaluation of hemorrhagic risk.

Management of malignant SVCS depends on symptom severity, tumor histology, anticipated response to systemic treatment, performance status, and expected survival [13,14]. Endovascular stenting can provide rapid relief in patients with severe or life-threatening obstruction, whereas chemotherapy and radiotherapy address the underlying malignancy in treatment-sensitive tumors [13,14]. Palliative thoracic radiotherapy remains a reasonable option when stenting is unavailable, unsuitable, or not selected and when reduction of the compressive or invasive tumor burden is required. Hypofractionated schedules using approximately 3-4 Gy per fraction over five to 10 fractions are commonly employed in patients with metastatic disease and limited prognosis, although no universally accepted optimal schedule exists [14]. A retrospective comparison in lung cancer-associated SVCS found that both 20 Gy in five fractions and 30 Gy in 10 fractions provided symptom relief, with the shorter regimen offering a practical option for patients with limited life expectancy [18]. Similarly, Patel et al. reported the use of urgent palliative radiotherapy (20 Gy in five fractions) for symptomatic SVCS secondary to PSC. Despite treatment, the patient's clinical condition continued to deteriorate, and he was ultimately transitioned to hospice care, reflecting the aggressive natural history of advanced disseminated PSC [12].

The patient also had two brain metastases, the larger of which was associated with vasogenic edema. Contemporary guidelines generally favor stereotactic radiosurgery for patients with a limited number of relatively small brain metastases and a reasonable performance status, whereas treatment should be individualized in patients with poor prognosis or severe extracranial disease [19,20]. In patients with impaired performance status, widespread systemic progression, or limited life expectancy, treatment should be individualized. When whole-brain radiotherapy is indicated for symptom palliation, short-course regimens, including 20 Gy in five fractions, remain widely accepted options in clinical practice [20]. In this case, the choice of 20 Gy in five fractions to both the whole brain and mediastinum minimized treatment duration while addressing cerebral disease and symptomatic SVCS.

Despite prompt initiation of corticosteroids, therapeutic anticoagulation, and emergency palliative radiotherapy, the patient's clinical condition deteriorated rapidly, and he died shortly after receiving the second fraction, before any clinically meaningful benefit from radiotherapy could reasonably be expected. This fulminant evolution was likely related to the combination of poor performance status, a large thoracic tumor, extensive multiorgan metastases, central vascular invasion, venous thrombosis, suspected tumor-related pulmonary embolism, and cerebral involvement. Advanced stage and distant metastasis are consistently associated with adverse survival in PSC [3,9]. The case therefore illustrates not only the aggressive biology of PSC but also the difficulty of achieving meaningful symptom relief when several immediately threatening complications are already present at diagnosis.

The diagnosis of PSC was established through the integration of the clinical presentation, cross-sectional imaging, bronchoscopic findings, histopathological examination, and immunohistochemical analysis. Although histopathological confirmation of the pulmonary arterial filling defect was not available, its radiological features, associated vascular invasion, and the overall clinicopathological context strongly supported a tumor-related etiology. This case highlights the highly aggressive biological behavior of PSC, which may present with multiple simultaneous oncologic emergencies, including malignant SVC syndrome, extensive venous thrombosis, brain metastases, and radiologically suspected pulmonary tumor embolism. It also underscores the importance of a multidisciplinary diagnostic approach integrating clinical, radiological, bronchoscopic, histopathological, and immunohistochemical findings to achieve an accurate diagnosis and guide appropriate management. Despite prompt supportive and palliative treatment, the patient's rapidly fatal clinical course illustrates the poor prognosis associated with disseminated PSC.

## Conclusions

PSC is a rare and highly aggressive malignancy that may present with multiple life-threatening oncologic emergencies at diagnosis. This case underscores the importance of integrating clinical, radiological, bronchoscopic, histopathological, and immunohistochemical findings to achieve a timely and accurate diagnosis in advanced disease. The coexistence of malignant SVC syndrome, extensive venous thrombosis, brain metastases, and radiologically suspected tumor-related pulmonary embolism illustrates the complex and life-threatening presentation of disseminated PSC and the need for prompt multidisciplinary management.
